# Setup Uncertainty of Pediatric Brain Tumor Patients Receiving Proton Therapy: A Prospective Study

**DOI:** 10.3390/cancers15225486

**Published:** 2023-11-20

**Authors:** Jared Becksfort, Jinsoo Uh, Andrew Saunders, Julia A. Byrd, Hannah M. Worrall, Matt Marker, Christian Melendez-Suchi, Yimei Li, Jenghwa Chang, Kavitha Raghavan, Thomas E. Merchant, Chia-ho Hua

**Affiliations:** 1 Department of Radiation Oncology, St. Jude Children’s Research Hospital, Memphis, TN 38105, USA; jinsoo.uh@stjude.org (J.U.); julia.a.byrd.med@dartmouth.edu (J.A.B.); hannah.worrall@stjude.org (H.M.W.); thomas.merchant@stjude.org (T.E.M.); chia-ho.hua@stjude.org (C.-h.H.); 2 Department of Biostatistics, St. Jude Children’s Research Hospital, Memphis, TN 38105, USA; 3 Department of Radiation Medicine, Donald and Barbara Zucker School of Medicine at Hofstra/Northwell, Hempstead, NY 11549, USA; 4 Department of Pediatric Medicine, St. Jude Children’s Research Hospital, Memphis, TN 38105, USA; kavitha.raghavan@stjude.org

**Keywords:** setup uncertainty, proton therapy, image guidance, pediatric patients, robust optimization, cone-beam CT, intrafractional patient motion, six degree-of-freedom

## Abstract

**Simple Summary:**

Proton therapy enables the delivery of a high radiation dose to tumors while sparing surrounding normal tissues. Inaccurate patient positioning may lead to underdosing of the targeted tumor and overdosing of nearby healthy tissues. Before a course of proton therapy, a CT scan is acquired for treatment planning with the patient in the treatment position, and this image dataset is used as a reference for patient localization at each treatment fraction. Radiation therapists perform daily cone-beam CT (CBCT) image guidance to align the patient to closely match the planning CT and minimize inaccuracies in radiation delivery. In this study, CBCT scans were systematically collected with clinical and treatment information to investigate questions related to image guidance, setup uncertainty, and patient motion during the treatment of pediatric brain tumors. Knowledge gained from this study provides a basis for designing safe and optimal proton treatments.

**Abstract:**

This study quantifies setup uncertainty in brain tumor patients who received image-guided proton therapy. Patients analyzed include 165 children, adolescents, and young adults (median age at radiotherapy: 9 years (range: 10 months to 24 years); 80 anesthetized and 85 awake) enrolled in a single-institution prospective study from 2020 to 2023. Cone-beam computed tomography (CBCT) was performed daily to calculate and correct manual setup errors, once per course after setup correction to measure residual errors, and weekly after treatments to assess intrafractional motion. Orthogonal radiographs were acquired consecutively with CBCT for paired comparisons of 40 patients. Translational and rotational errors were converted from 6 degrees of freedom to a scalar by a statistical approach that considers the distance from the target to the isocenter. The 95th percentile of setup uncertainty was reduced by daily CBCT from 10 mm (manual positioning) to 1–1.5 mm (after correction) and increased to 2 mm by the end of fractional treatment. A larger variation existed between the roll corrections reported by radiographs vs. CBCT than for pitch and yaw, while there was no statistically significant difference in translational variation. A quantile mixed regression model showed that the 95th percentile of intrafractional motion was 0.40 mm lower for anesthetized patients (p=0.0016). Considering additional uncertainty in radiation-imaging isocentricity, the commonly used total plan robustness of 3 mm against positional uncertainty would be appropriate for our study cohort.

## 1. Introduction

The sharp dose gradient of pencil-beam scanning proton therapy provides a desirable dose distribution highly conformal to the target volume while sparing healthy tissues and lowering the secondary malignancy risk by reducing the integral dose [1,2,3]. This rapid dose fall-off comes at a cost because high accuracy and precision are crucial in daily patient setup. Differences in the target (i.e., patient) position between treatment plans and actual treatment sessions, known as setup error, can result in suboptimal dosage, which negates the advantage of proton beams [4,5,6]. Underdosing of the tumor itself can lead to inadequate coverage and an increased likelihood of marginal recurrence, while a high dose delivered to adjacent critical organs can lead to treatment toxicity. This is especially relevant for children receiving radiotherapy [7] because the distance between the target and critical organs is often shorter than that of adults and the longer posttreatment lifespan of childhood cancer survivors presents more risk for the manifestation of late adverse effects. Developing pediatric tissues are also thought to be more sensitive to radiation than adult tissues, which may increase the likelihood of toxicities and secondary cancers [8,9,10,11,12].

Setup uncertainty for adult and pediatric brain tumor patients receiving image-guided photon therapy have been reported to be 1 to 2 mm in each translational direction (lateral, longitudinal, and vertical) and usually less than 1 degree for each angle of rotation (pitch, roll, and yaw) [13,14,15,16]. Data for patients receiving image-guided proton therapy on a modern robotic 6 degree-of-freedom (DOF) couch are lacking in the current literature. Patient setup errors are not considered medical errors per se; however, large errors are undesirable. Photon therapy traditionally accounts for day-to-day variation in treatment position by expanding the clinical target volume (CTV) in all directions to create planning target volume (PTV) [17,18]. Pretreatment image-guided correction is employed to reduce the need for a large margin, which can be up to 5 mm [19,20,21]. Daily imaging reduces the uncertainty in the position more significantly than weekly imaging does, and therefore permits a smaller margin [15]. The concept of the PTV margin was proposed for photon therapy based on the assumption that shifts in patient position are not expected to significantly change the dose distribution (i.e., shift invariance) [2]. However, this assumption rarely applies to proton therapy [22,23]. Instead of building the setup uncertainty into the PTV margin, robustness of a proton therapy plan is achieved by incorporating high but plausible error scenarios of patient setup and proton range uncertainties into the optimization process. These error scenarios or perturbations are generated based on user-specified, site-specific uncertainty settings by shifting the beam isocenter (e.g., ±3 mm) along three orthogonal axes and simultaneously scaling the CT numbers (e.g., by ±3–3.5%) [2,24,25]. Rotational perturbation is not available in commercial robust optimization.

In this work, we report findings from a prospective imaging trial that quantifies daily setup errors and intrafractional movements of pediatric patients with brain tumors undergoing proton therapy with volumetric image guidance of 6 DOF robotic couch corrections. Setup errors from manual positioning by therapists and residual errors after image-guided corrections were compared to demonstrate the value of image guidance. We incorporated both translational and rotational errors into the calculation of appropriate robustness settings using a statistical simulation approach. Effects of anesthesia and sedation on intrafractional motion were examined. These pediatric-specific data, previously unreported in the literature, provide the basis for knowledge-based robust optimization and evaluation in the contemporary setting of image-guided proton therapy.

## 2. Methods and Materials

### 2.1. Prospective Protocol and Patient Cohort

An Institutional Review Board approved the prospective, non-therapeutic imaging protocol (ClinicalTrials.gov ID: NCT04125095) to quantify the setup uncertainty and patient movement in pediatric proton therapy opened in 2020. A total of 183 patients were enrolled in the head cohort, which included patients treated for tumors in the head but excluded data from craniospinal irradiation (CSI) cases because the setup procedure differs between the two. Enrollment is ongoing for the body cohort, and setup uncertainty for body and CSI patients will be analyzed and reported in a future publication. Eighteen enrolled head cohort patients were excluded from this study after enrollment, leaving 165 for analysis (Table 1). Special conditions resulted in the exclusion of 6 patients: 3 that were uncooperative during setup, 2 that were over 25 years old, and 1 patient that ultimately only received spinal irradiation during his treatment course. A total of 12 patients were removed due to machine downtimes, image capture errors, or physician or parent discretion for the patient to be unenrolled from the study.

### 2.2. Imaging System and Protocol Workflow

Imaging systems used to gather the data for this protocol were described in a previous publication [26]. Briefly, a ceiling-mounted robotic cone-beam CT (CBCT) system sends reconstructed images to commercial image registration software PIAS (v5.3.0, Hitachi Ltd., Tokyo, Japan), which calculates a 6-parameter setup correction (3 translations and 3 rotations) for a 6 DOF robotic patient positioner to implement, and 3D-to-3D image registration was first performed automatically using a mutual information algorithm and then reviewed (and adjusted manually if necessary) by therapists. The CBCT system can acquire kilovoltage volumetric imaging or orthogonal radiographs at the treatment isocenter of the half gantry proton therapy system. When orthogonal radiographs were acquired, 3D-to-2D registration was performed by matching a series of digitally reconstructed radiographs synthesized from planning CT to acquired 2D images. All imaging and proton therapy systems were commercial equipment by Hitachi Ltd.

**Figure 1:**
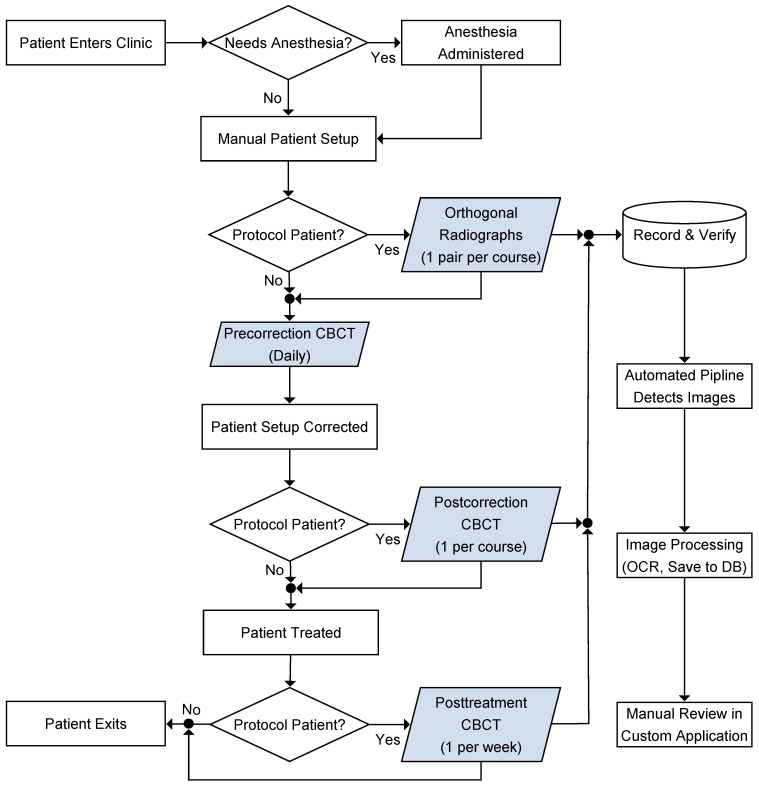
Clinical and analytical workflow for protocol. Shaded (highlighted in blue) boxes represent imaging data for analysis. OCR: Optical character recognition. DB: database.

The workflow of image acquisition and processing for protocol patients is illustrated in Figure 1. CBCT was acquired daily after initial setup on the treatment couch by therapists. Daily pretreatment CBCT image guidance is standard of care in our clinic, irrespective of protocol participation. Research imaging for protocol patients included one-time orthogonal radiographs acquired immediately before pretreatment CBCT for paired comparisons of planar and volumetric image guidance, one-time repeat CBCT to measure residual errors after implementing the calculated correction from pretreatment CBCT (thereafter called postcorrection CBCT), and CBCT acquired weekly immediately after fractional beam deliveries while patients were still in the treatment position to estimate the intrafractional patient movement (posttreatment CBCT). The standard daily pretreatment CBCT is called precorrection CBCT in this study to distinguish from the postcorrection CBCT. Research imaging was not acquired daily to reduce imaging dose to patients and time spent in the treatment room.

**Figure 2:**
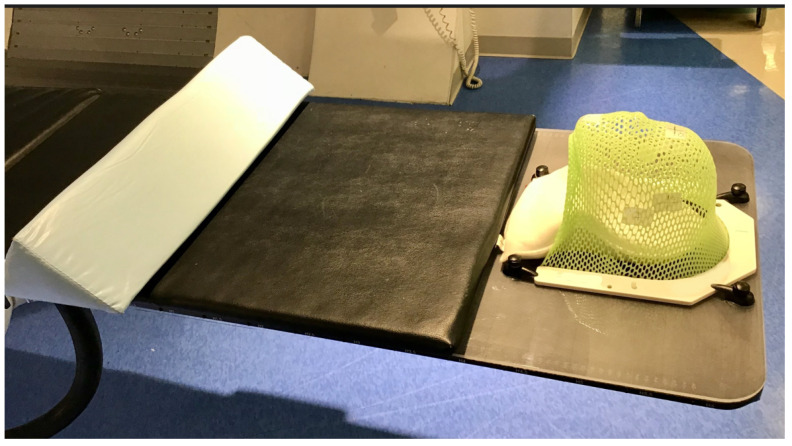
To reduce setup error and intrafractional motion, a full head thermoplastic mask with a U-frame was placed over the patient’s face and fixated to an indexed polycarbonate overlay board latched to the tabletop. A triangle sponge cushion was placed under the patient’s knees.

All patients were in supine position, with customized head and neck support. During the workflow, therapists ensured that the patient’s head was straight, and chest and shoulders were centered along the mid-sagittal plane using lasers to check alignment to suprasternal notch. A full-head thermoplastic mask with a U-frame was placed over the patient’s face and fixated to an indexed polycarbonate overlay board latched to the tabletop (Figure 2). Attention was given to confirm that the forehead, chin and bridge of nose were articulating with the mask to ensure a proper mask fit. If sedated or anesthetized, the team would confirm that the patient’s airway was unobstructed and could tolerate the position for the duration of the treatment. Therapists then adjusted the table for 3-point markings on the mask and a sagittal alignment mark on the chin to align with room lasers. A safety belt was secured around the patient’s abdomen. Propofol-based total intravenous anesthesia was the regimen of choice with or without supplemental anesthetic drugs for young children. Moderate sedation was used as needed when patients did not require general anesthesia.

### 2.3. Automatic Data Extraction and Database Entry

**Figure 3:**
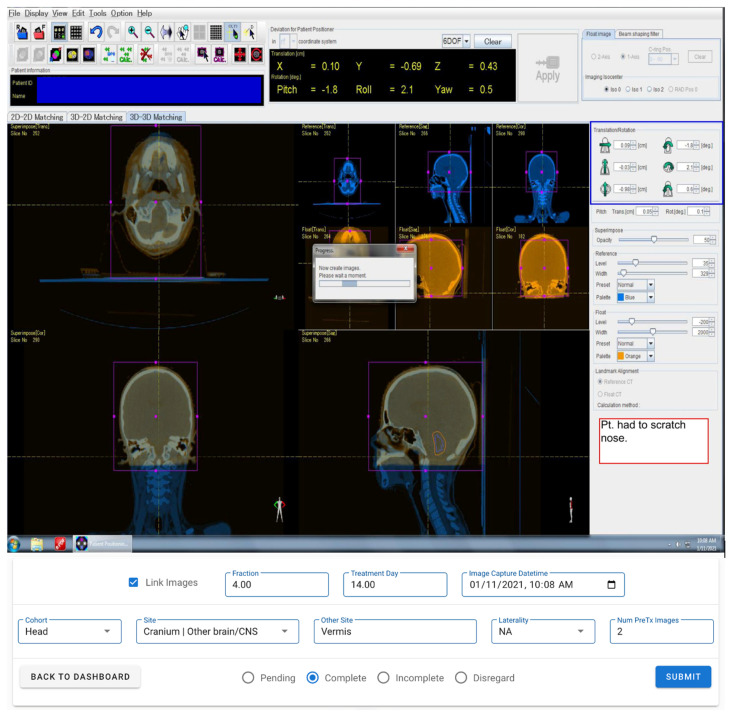
An example screen capture of Patient Positioning and Image Analysis (PIAS) software (v5.3.0), stored for each patient CBCT scan in our custom data management and manual review application. Setup corrections or residual errors (blue boxes) in CBCT coordinates and special conditions (red box) entered by therapists are captured using optical character recognition. Fractional and cohort information are queried from record and verify system queued for manual review in the application (form inputs at bottom).

For each imaging study, several data values were required to be captured and entered into a clinical trial database. They included a numerical value for each of the 6 DOF setup correction shifts and rotations calculated by the imaging registration software, treatment day, fraction number, assigned cohort, treatment site, image capture timestamp, and any relevant special conditions documented by treating therapists (Figure 3). We developed an automated pipeline and application to improve the efficiency of the data collection process and increase the accuracy of the entered values. First, the pipeline detected the presence of new images in the record and verify system (MOSAIQ, Elekta Solutions AB, Stockholm, Sweden). Python scripts were written to process the screen capture, and optical character recognition was used to extract numerical values (calculated setup corrections or residual errors) and special condition texts entered by therapists from each image dataset (Tesseract v5.0). A PostgreSQL (v13.4) database was developed to store the information, and a custom web application queued processed images for manual review by clinical research associates, who could correct data if needed within the application itself. Only data which were manually reviewed and approved by trained staff were included in this study.

### 2.4. Including Both Translations and Rotations into Setup Uncertainty Calculations

**Figure 4:**
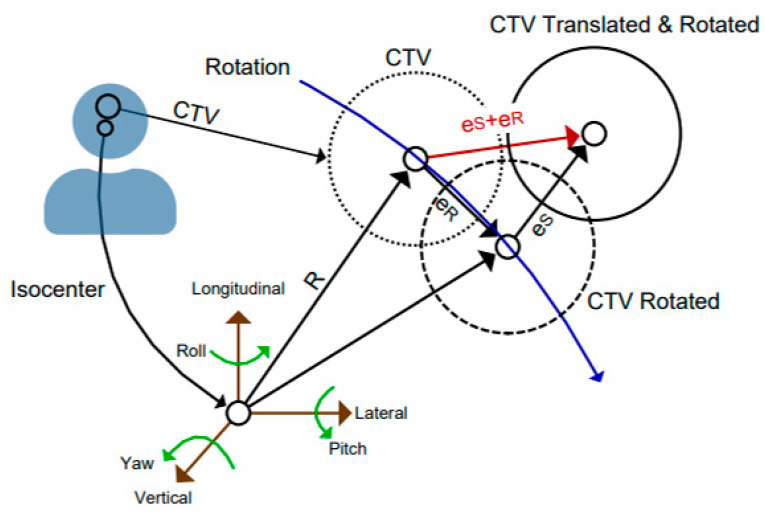
There are six degrees of freedom of movement about the treatment isocenter. Translation and rotation should be considered when determining the setup error. R is the distance between CTV and treatment isocenter. **e_R_** is the displacement after rotational correction. **e_S_** is the translational correction.

Modern treatment couches have 6 DOFs of motion: 3 translational and 3 rotational. Implementing both translational and rotational corrections about the treatment isocenter would improve the reproducibility of patient position from conventional corrections by 3 translations only (the coordinate system adopted in this study is illustrated in Figure 4). However, it is challenging to implement the extra rotational motion into current commercial treatment planning systems which only simulate perturbations in 3 translational axes. The challenge particularly arises from the fact that rotational motions induce different translational displacements depending on the specific location of the target respect to the origin of the rotation, which is typically the treatment isocenter. While it is desirable to place the treatment isocenter within the CTV, it is not always possible. For tumors located laterally (away from the midline), the patient table needs to be laterally shifted to place the CTV at the isocenter, which could increase the risk of collision between the tabletop and the CBCT C-ring for our proton system. Additionally, for patients with multiple metastases or multifocal tumors, it would not be possible to locate the isocenter at the centroid of each CTV body without repositioning the patient and between beam deliveries and complicating treatment planning.

A new PTV margin recipe incorporating rotational information has been explored by utilizing a statistical approach [27,28], which alleviates the need for information of the patient-specific target location other than the distance from the isocenter. In the present study, we extended the statistical approach for robust planning in proton therapy to allow the 6 distributions of the setup errors (one for each DOF) to have unequal variances (Supplementary Materials). The distribution of the full error vector ($e_{S}+e_{R}$ in Figure 4), which considers both translational and rotational errors, can be sampled from the separate distributions, converting a deterministic six-dimensional problem into a stochastic three-dimensional problem (Algorithm 1). The variances of the converted three-dimensional movement are dependent on the distance from the origin of rotation, and they are larger than the 3 translational variances, reflecting the extra uncertainties originated from rotational movements. The 3 independent normal distributions will in general have different variances, so Monte Carlo sampling of the 3 independent normal distributions is required to estimate the means, 95th percentiles, and confidence intervals for the Euclidean norm of the full error vector. This sampling converts the three-dimensional problem into a problem of sampling scalars which represent the length of the full error vector, enabling comparisons of uncertainty between 6 DOF movement problems with one-dimensional robust optimization setting parameters in commercial treatment planning systems.
**Algorithm 1: Draw one sample from the 3D problem that considers all 6 degrees of freedom**Let R be the distance from the treatment isocenter to the centroid of the CTV. Let x,y, and z be the lateral, longitudinal, and vertical components of the patient setup error vector. Let α, β, and γ represent yaw, roll, and pitch, respectively, of the same vector.# This depends on the rotation order during position correction and can vary by machine. Let $\sigma_{x}^{2},\;\sigma_{y}^{2},\;\sigma_{z}^{2},\;\sigma_{\alpha }^{2},\;\sigma_{\beta }^{2},\;\sigma_{\gamma }^{2}$ be the variances of each degree of freedom across the cohort. Transform 6 variances into 3 (one for each translational direction): $\bar{p_{x}^{2}}=\frac{R^{2}}{3}\left(\sigma_{\beta }^{2}+\sigma_{\gamma }^{2}\right)+\sigma_{x}^{2}$              # Total variance in lateral direction.$\bar{p_{y}^{2}}=\frac{R^{2}}{3}\left(\sigma_{\alpha }^{2}+\sigma_{\gamma }^{2}+\sigma_{\alpha }^{2}\sigma_{\beta }^{2}+\sigma_{\alpha }^{2}\sigma_{\beta }^{2}\sigma_{\gamma }^{2}\right)+\sigma_{y}^{2}$      # Total variance in longitudinal direction.$\bar{p_{z}^{2}}=\frac{R^{2}}{3}\left(\sigma_{\alpha }^{2}+\sigma_{\beta }^{2}+\sigma_{\alpha }^{2}\sigma_{\gamma }^{2}+\sigma_{\beta }^{2}\sigma_{\gamma }^{2}\right)+\sigma_{z}^{2}$        # Total variance in vertical direction. Draw from 3 normal distributions ($N\left(\mu ,\sigma^{2}\right)$) to obtain one sample from the error $e_{S}+e_{R}$ in Figure 2. $x_{new}\;\sim \;N\left(x,\;\bar{p_{x}^{2}}\right)$, $y_{new}\;\sim \;N\left(y,\;\bar{p_{y}^{2}}\right)$, $z_{new}\;\sim \;N\left(z,\;\bar{p_{z}^{2}}\right)$  # Each is a component of a 3D vector. Return $\sqrt{x_{new}^{2}+y_{new}^{2}+z_{new}^{2}}$              # Length of sampled vector.

### 2.5. Statistical Analysis and Simulations

Individual degrees of freedom were investigated independently by generating histograms for the displacement of each image type (precorrection, postcorrection, and posttreatment CBCT). Three comparisons were made between orthogonal and CBCT imaging. First, paired exact Wilcoxon signed-rank tests with continuity correction were run for each degree of freedom individually to determine if the 2D setup error differed from the 3D setup error. *p*-values were adjusted for multiple hypothesis correction using the Holm method, which is less conservative than the Bonferroni adjustment but is still valid under arbitrary assumptions [29]. Second, 2D minus 3D was calculated for the DOF of each patient, and F-tests were run to determine if there were differences in variance for the resulting difference distributions. Translational and rotational DOFs were not compared against each other, and all pairs of the same units (mm vs. mm and degrees vs. degrees) were tested. Shapiro–Wilk tests for normality were conducted to ensure validity of the F-test for each difference distribution. Third, simulations of Algorithm 1 were carried out using the matched orthogonal and CBCT image pairs to estimate the differences in setup corrections suggested by each modality as a function of the distance between target volume and isocenter.

Overall (translation and rotational) postcorrection and intrafractional errors were studied by multiple simulations. The effects of the distance between the isocenter and CTV from R = 0 to 10 cm were accounted for in the Monte Carlo simulation of Algorithm 1 to generate the 95th percentile and its percentile bootstrap confidence intervals. The overall setup error was simulated 2000 times for each R value. For each timepoint (daily pretreatment/precorrection, postcorrection, weekly posttreatment) of each patient, a representative overall error vector was calculated by running Algorithm 1 1000 times for each measured patient setup error, yielding an estimate of the setup error which can be compared across timepoints to understand variation in setup within treatment fractions. The variances were recalculated for each bootstrap sample. A linear mixed-effects model [30] was constructed using the sampled error lengths to compare the mean intrafractional motion of patients receiving anesthesia or sedation vs. patients that did not receive anesthesia or sedation. Mixed modeling was used to account for repeated measurements for each patient, missing data for some timepoints, and because patient and timepoint are random effects, while anesthesia was a fixed effect. Linear quantile regression models were fit to study the differences between the 95th percentile of intrafractional motion based on anesthesia status [31,32].

## 3. Results

A total of 3737 precorrection CBCT scans from 165 patients were acquired. For 40 patients, a single paired 2D orthogonal X-ray image was acquired. Protocol statistical stopping criteria suggested that this would have sufficient power for 3D-to-2D comparison. Each of 145 patients received a single postcorrection CBCT scan, and 161 patients received a total of 681 posttreatment CBCT scans. The numbers of patients for each CBCT type are not equal because not all patients received their postcorrection CBCT or posttreatment CBCT for every week due to machine downtimes or scheduling conflicts in the clinic.

**Figure 5:**
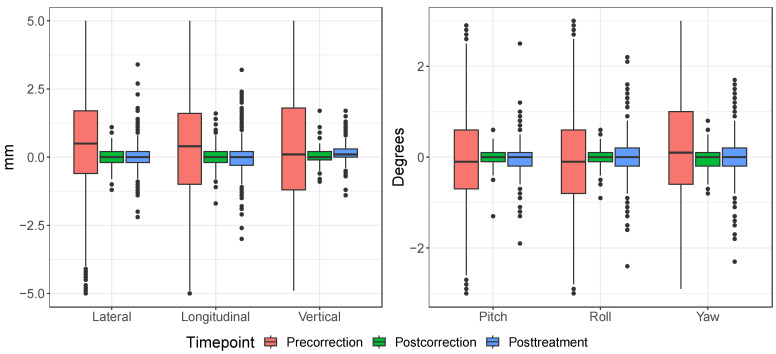
Distributions of setup error for each degree of freedom and time point. Some extreme outliers are not shown in the plot.

**Figure 6:**
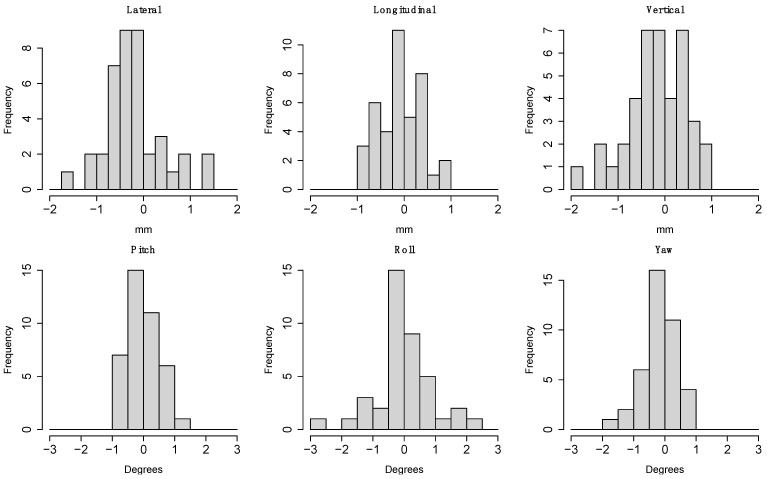
Histograms of difference between corrections suggested by orthogonal X-ray (2D) and CBCT (3D). Roll has significantly higher variance (F-test; normality checked) than pitch and yaw (Holm-corrected *p*-values 0.0002 and 0.0129, respectively).

**Figure 7:**
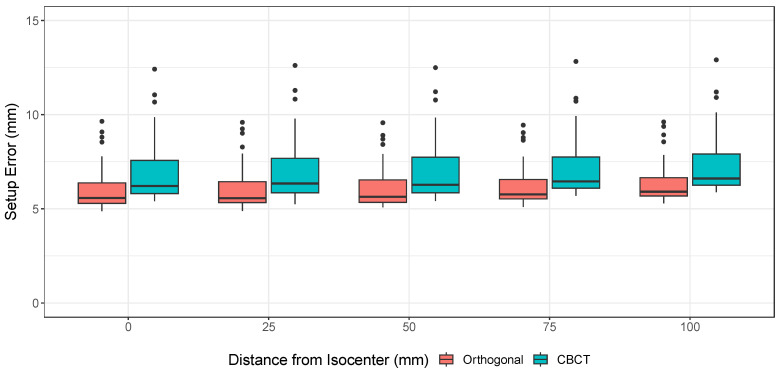
Orthogonal X-ray and CBCT setup error simulations show that there is a significant difference (0.78 mm, mixed model, p=0.012) between the corrections suggested by the two methodologies.

The individual distributions of setup error for each DOF determined by CBCT are shown by timepoint in Figure 5. As can be seen, CBCT guidance significantly reduced the setup errors from precorrection to postcorrection, although the distributions broaden slightly by the end of fractional treatments. The comparison for each individual dimension between orthogonal X-ray and CBCT (orthogonal minus CBCT for all 40 paired images) is shown in Figure 6. Each distribution passed normality checks using the Shapiro–Wilks test, so the F-test was considered a valid test for differences in variance. Holm-corrected hypothesis tests showed that there was significantly larger variance between the roll dimension and the other rotational dimensions pitch and yaw (corrected *p*-values of 0.002 and 0.0129, respectively). None of the translational dimensions showed significant differences in variance after multiple hypothesis correction. Simulation results show a statistically significant difference of 0.78 mm (mixed model, p=0.012) between orthogonal and CBCT setup errors (Figure 7). The smaller setup errors of orthogonal images indicate that the associated position corrections were limited, compared to the more extensive corrections by CBCT, suggesting an undercorrection.

**Figure 8:**
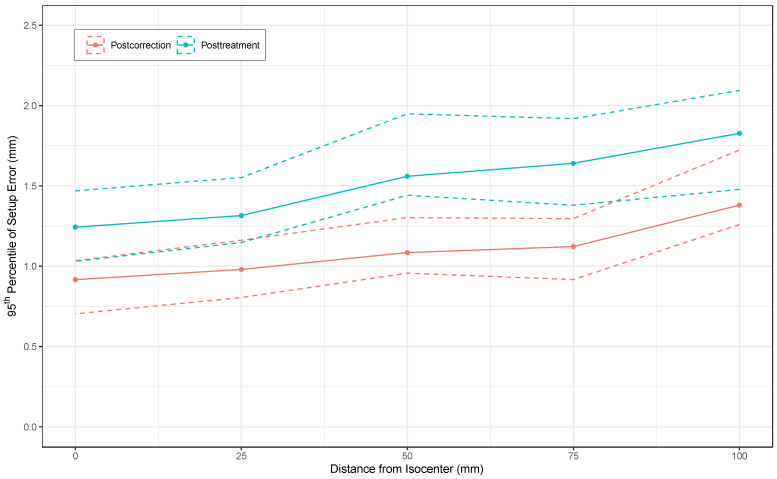
The 95th percentiles (solid lines) and Studentized bootstrap confidence intervals (dashed lines) for total setup error (translational and rotational) based on target distance from treatment isocenter.

**Figure 9:**
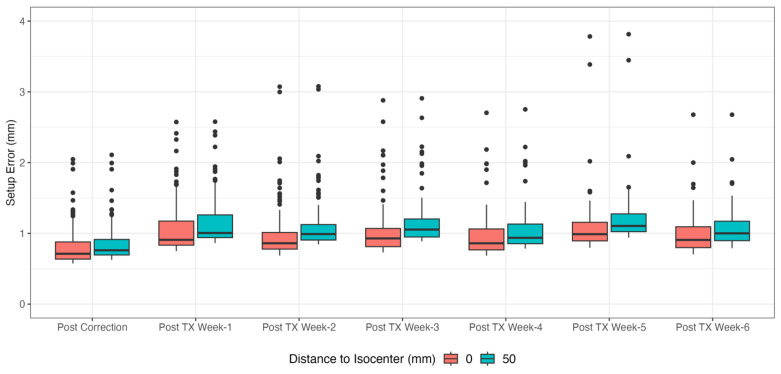
Total setup error based on timepoint and simulated distance from isocenter. Posttreatment error (intrafractional motion) is higher than postcorrection error (linear quantile mixed model, p<0.0001), but the 95th percentiles do not exceed 2 mm.

The postcorrection and posttreatment setup errors increase with distance from treatment isocenter (R) to CTV (Figure 8). A total of 500 bootstrap samples of the 95th percentile were taken for each R from 0 to 100 mm in increments of 25 mm. Postcorrection and posttreatment setup errors are shown by treatment week in Figure 9. A linear quantile regression mixed model was fit with the response as the 95th percentile and the fixed effect being postcorrection vs. posttreatment, with repeated measures allowed per patient (weeks 1 to 6), and the patient as a random effect. The fitted model indicated that the posttreatment 95th percentile was 0.31 mm higher than the postcorrection error (p<0.0001). Another linear mixed-effects model fit to the mean rather than the 95th percentile showed that the mean error is 0.22 mm higher posttreatment (p<0.0001).

**Figure 10:**
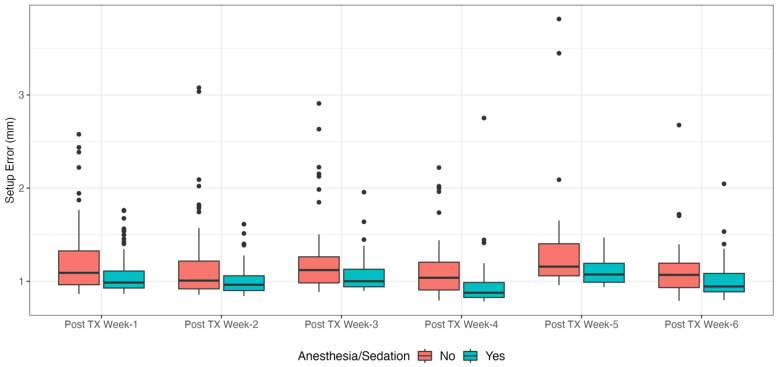
Patient receiving anesthesia or sedation have significantly lower (0.17 mm) mean intrafractional motion (mixed model, p<0.0001). The 95th percentile is 0.40 mm lower (linear quantile mixed model, p=0.0016).

The effects of anesthesia/sedation are displayed in Figure 10. Patients receiving anesthesia or sedation (median age: 5 years, range: 0 to 21) have significantly lower (0.17 mm) mean intrafractional motion (mixed model, p<0.0001) than awake patients (median age: 12 years, range: 6 to 24). The 95th percentile is 0.40 mm lower (linear quantile mixed model, p=0.0016).

## 4. Discussion

In the era of 6 DOF robotic patient positioners and daily volumetric image guidance, we have demonstrated how to conduct a prospective study to systematically collect and analyze imaging data acquired at different timepoints throughout the course of proton therapy. Results provide a quantitative basis for designing the appropriate robustness for pediatric brain tumor patients receiving proton therapy and allow for the identification of outliers for further investigation and process improvement. The medians and 95th percentiles of the individual DOF of the setup errors greatly decreased from precorrection with manual positioning to postcorrection and posttreatment timepoints, clearly demonstrating the benefits of image guidance in pediatric radiotherapy (Figure 5). Furthermore, no bias was observed in any of the DOFs after correction. There was a slight increase in posttreatment positional deviation due to intrafractional motion despite the mask immobilization. However, it is reassuring to confirm that the 95th percentiles were still well within 3 mm, a number our center and many others use daily in robust optimization and evaluation when designing proton plans for cranial tumors.

Our institution previously quantified the setup uncertainty with megavoltage CBCT for 100 children treated for brain tumors or head and neck cancers with photon therapy on a 3 DOF couch [15]. A setup margin of 2 mm was determined to be appropriate for supine patients with daily CBCT guidance based on both precorrection and posttreatment CBCT data and the geometric margin formula developed by van Herk et al. [33]. Our current study investigated a larger cohort of pediatric proton patients receiving daily modern 6 DOF volumetric image guidance. A rigorous statistical analysis estimated that the 95th-percentile positional accuracy of 2 mm can be maintained by the end of fractional treatments. In comparison with adult studies, Kanakavelu et al. derived a geometric margin of 3.5 mm, 2.4 mm, and 1.9 mm in the lateral, longitudinal, and vertical directions for brain tumor patients receiving mixed megavoltage planar and volumetric image guidance [34]. Zechner et al. calculated the margin to be 0.8 mm, 1.2 mm, and 0.6 mm, respectively, for adult head patients and 0.8 mm, 0.5 mm, and 0.9 mm, respectively, in anesthetized pediatric head patients based on intrafractional displacement data extracted from orthogonal radiographs and 2D-3D registration [35]. It should be noted that many prior studies have been limited to 3 DOF image registration and couch corrections for which unmeasured rotational errors could impact estimated translational setup uncertainty. They may assume image-guided corrections implemented by couch movement can completely remove the setup errors, unlike our approach, which measured the residual errors with postcorrection CBCT and intrafractional movement with posttreatment CBCT. Additionally, institution-specific setup procedures, immobilization devices, setup correction schemes/thresholds, and clinical judgement all affect the determination of target margin and robustness. Comparisons across studies should be performed with these considerations in mind.

Many proton centers continue to utilize daily orthogonal radiographs for guiding pediatric cranial setup rather than CBCT due to the considerations of speed and reduced radiation exposure. Therefore, our data are of value to those determining the tradeoff between setup accuracy and time/exposure. There were no significant differences between the medians of the errors of the individual dimensions, but the higher roll variance compared to pitch and yaw (Figure 6) demonstrates the utility of CBCT over orthogonal X-ray for correcting the head rotation about the longitudinal axis. Inaccurate roll measurements reported by orthogonal X-ray can affect the accuracy of 2D-to-3D image registration between X-ray and planning CT [36]. Because of the inability to visualize and adjust roll rotations easily with orthogonal X-ray, rotational errors may be undercorrected, leaving residual errors that can decrease treatment accuracy. This is especially important for patients with tumors of irregular shape, with immediately tumor-adjacent critical structures, or that are not centered on the treatment isocenter. When the total calculated setup errors were compared between the two image guidance modalities (Figure 7), the difference was statistically significant. Our data suggest that planar image guidance might have undercorrected setup errors by 0.78 mm had it been used in our clinic. The smaller correction suggested by orthogonal imaging likely resulted from the less accurate calculation of patient roll errors (Figure 6).

Figure 8 shows that setup uncertainty increases with distance from the treatment isocenter. This relationship is quadratic, as seen in the total variance equations in Algorithm 1, but at the smaller distances common in treatment of pediatric brain tumors, the increase is roughly linear because the result is dominated by the cohort variances ($\sigma_{x}^{2},\sigma_{y}^{2},\;\sigma_{z}^{2}$) for smaller R. When the isocenter and CTV centroid are identical (R = 0), the estimated postcorrection and posttreatment setup error confidence intervals include 1 mm but not 2 mm, suggesting that the 2 mm robustness for setup error is adequate for treatment planning. The upper confidence intervals of the 95th percentiles of the combined translational and rotational postcorrection setup error and intrafractional motion are both lower than 2 mm for all practical intracranial distances. Therefore, we conclude that the 2 mm assumption for translation and rotational error is adequate for patients after positional correction by a 6 DOF robotic couch. While it would be unusual for the CTV centroid of an intracranial patient to be further than 10 cm from the treatment isocenter due to the limited head size, our algorithm is generalizable to other body sites in which such distances are plausible. It is important to note, however, that these simulations calculate variance based on bootstrap resamples of the postcorrection cohort from our institutional dataset. The generalizability of this specific result to other clinics will depend on the accuracy of the image-guided correction by the robotic couch and clinical practice of therapy staff. It should be noted that the variances in Algorithm 1 are sensitive to outliers arising from special conditions of patients and potential data entry errors. We accounted for this by documenting special conditions for each fraction and manually reviewing the results of the automated data extraction with our custom application.

The posttreatment errors, reflecting intrafractional motion, shown in Figure 9, are consistent across the entire treatment course. The difference in the 95th percentile estimated by the linear mixed quantile regression model indicates that motion during treatment is nonzero but still within the robustness optimization setting. The 0.28 mm difference between values at R = 0 cm and R = 5 cm (Figure 9) suggests the contribution of rotational uncertainty to the setup error is small for typical intracranial distances.

Our study findings on intrafractional motion did not indicate the need for additional imaging between beam delivery within a treatment fraction, unlike the conclusion from a previous adult head and neck study [37]. They reported a trend of increasing intrafractional positional deviations toward the end of treatment course and a positive correlation with body mass index. Only when patients had a body mass index <27 kg/m^2^ and no weight loss was kV imaging not required following a couch rotation. In our head cohort, the 95th percentile of the total setup error calculated from posttreatment CBCT was within 2 mm for both awake and anesthetized patients. Due to the half-gantry design and the frequent use of noncoplanar beams, treatment of our brain tumor patients often requires large angle couch rotations. However, these couch movements did not appear to disturb patient positions to a point that requires intrafractional readjustment. We suspect that a few outliers with larger intrafractional motion may be related to the poorly fitting mask from on-treatment changes in hair style/loss or skin surface. Some may be due to patient cooperation or discomfort. Further investigation is needed to better predict extreme outliers for increasing the patient comfort and improving the immobilization process.

Kilovoltage CBCT for image guidance could deposit a higher dose to critical organs in young children compared to adults if the same CBCT scan protocols are used [38]. Deng et al. reported a factor of 2 to 3 (higher to bones) with 125 kVp CBCT in half fan mode based on their Monte Carlo simulation [39,40]. It is essential to ensure that the daily use of CBCT for children does not overexpose patents and the benefits outweigh the risks. For each of our patients, we selected a proper scan protocol (kVp, mAs, half fan vs. full fan, and angular range) based on the imaged anatomy and body size and used collimation wherever feasible. The measured CT dose index (CTDIvol), a weighted average measure of radiation dose to representative head and body phantoms, of this CBCT system is comparable to what have been reported in the literature for modern CBCT systems used in photon radiotherapy and significantly lower than the doses from diagnostic quality CT scans and mega-voltage CBCT scans. As an example, one of our head protocols uses 100 kVp/15 mA/20 ms/200° rotation setting, which results in 2.7 mGy of CTDIvol. This translates to a total imaging dose of 81 mGy for standard patients or 100 mGy for research protocol patients over a treatment course of 30 fractions. We do not expect the imaging dose to significantly increase the risk of normal tissue complication even for radiation-sensitive organs, such as lens. To put into perspective for radiation therapy patients, typical delivered radiation doses to brain tumors are 54–55 Gy (54,000–55,000 mGy). The total radiation dose from daily CBCT guidance is approximately 0.2% of the therapeutic dose, which is well below the AAPM TG-180 threshold of a 5% therapeutic dose for considering in the treatment planning process [41].

This study has a few limitations. First, we relied on weekly posttreatment CBCT to capture intrafractional motion. Because they were acquired at the end of fractional treatments, we were not able to determine if patients moved during beam delivery or between the end of beam delivery and CBCT imaging. Second, comparisons of patient movements in anesthetized/sedated and awake patients were performed on two groups of patients with different age distributions. The effects of anesthesia and sedation on intrafractional motion may have been underestimated because the patients that physicians prescribed to receive anesthesia would likely have moved more during treatment than patients for whom anesthesia was deemed not necessary. Finally, this study did not include dosimetric analysis to assess the impact of less accurate setup corrections based on orthogonal radiographs, especially in the roll direction. The dose perturbation is likely case-dependent on the beam path heterogeneity. When critical organs such as lens, optic nerves, and brainstem are adjacent to the target, we anticipate that daily volumetric image guidance would best preserve plan quality over the treatment course. This study confirms that a 2 mm setup error margin is acceptable for pediatric proton therapy. The 3 mm robustness setting includes this 2 mm for patient setup uncertainty and an additional 1 mm for other uncertainties. Other important sources of uncertainty to consider in the treatment planning process, which we did not investigate in this study, include uncertainties in target delineation, dose calculation accuracy, proton-beam spot position, gantry and couch rotation, and imaging system alignment to the treatment beam. More research on these sources of uncertainty would need to be conducted before a recommendation could be made to increase/decrease the overall robust optimization settings.

## 5. Conclusions

Our prospective study of pediatric cranial setup uncertainty demonstrated that the 95th percentile can be reduced to approximately 1 mm with daily CBCT guidance and the treatment isocenter located near the CTV centroid. While patient motion under an immobilization mask increased that value to 2 mm by the end of fractional treatments, the commonly adopted 3 mm robustness setting for setup uncertainty would still provide a small buffer against additional system uncertainty such as radiation-imaging isocentricity. Compared to setup corrections calculated from CBCT, orthogonal radiographs showed a significantly larger variance in the roll rotation, indicating the challenge in 3D-2D registration with planar X-rays. Anesthesia reduced intrafractional cranial motion in pediatric patients to below 2 mm in almost all fractions compared to 3 mm in those awake.

## Figures and Tables

**Table 1:** Patient demographic characteristics and imaging studies analyzed.

	Patients	Count (%)
Enrollment	Total	183
	Included for Analysis	165
Age (years)	0 to 5	45 (27)
	6 to 10	56 (34)
	11 to 15	40 (24)
	16 to 20	16 (10)
	21 to 25	8 (5)
Sex	Male	99 (60)
	Female	66 (40)
Race	White	130 (79)
	Black	18 (11)
	Asian	9 (5)
	Multiple	7 (4)
	Unknown	1 (0.6)
Diagnosis	Medulloblastoma	45 (27)
	Craniopharyngioma	41 (25)
	Astrocytoma	18 (11)
	Ependymoma	17 (10)
	Atypical Teratoid Rhabdoid Tumor	8 (5)
	Germinoma	6 (4)
	Other	30 (18)
Anesthesia	Awake	85 (52)
	Anesthetized	77 (47)
	Sedated	3 (2)
	**Imaging Studies**	
Precorrection	Imaging Studies	3737
	Patients	165
Orthogonal Radiographs	Imaging Studies	40
	Patients	40
Postcorrection	Imaging Studies	145
	Patients	145
Posttreatment	Imaging Studies	681
	Patients	161

## Data Availability

The data presented in this study are available on request from the corresponding author. The data are not publicly available due to patient privacy requirements.

## Supplementary Materials

The following supporting information can be downloaded at: https://www.mdpi.com/article/10.3390/cancers15225486/s1.
