# Supplementary material for: Setup Uncertainty of Pediatric Brain Tumor Patients Receiving Proton Therapy: A Prospective Study
Source: Cancers (Basel). 2023 Nov 20;15(22):5486. doi: 10.3390/cancers15225486 (PMC10670653; doi:10.3390/cancers15225486)
Supplement: Supplementary file 1 [file cancers-15-05486-s001.zip › cancers-2677858-supplementary.pdf]

Appendix A

This appendix is to describe the procedure of determining uncertainty margin from the group data of 6 degrees of set up errors (translations in lateral, longitudinal, and vertical directions and rotations of pitch, roll, and yaw). Since the displacement induced by rotation depends on the distance from the axes and origin of the rotations, we consider a point in a 3-dimensional space, which may represent the location of the tumor of interest.

A.1 Individual disablements by setup errors

Let a column vector $\mathbf{p} = [p_x \; p_y \; p_z]^T$ be the intended location of the point. The subscripts x, y, and z represent the lateral, longitudinal, and vertical coordinates from the origin, respectively. Suppose this point is misplaced at $\mathbf{p}' = [p'_x \; p'_y \; p'_z]^T$ by the 6 degrees of set up errors. This displacement can be defined by a rotational matrix, $\mathbf{R}(\alpha, \beta, \gamma)$, and an orthogonal translational vector $\mathbf{d} = [d_x \; d_y \; d_z]^T$:

$$\mathbf{p}' = \mathbf{R}(\alpha, \beta, \gamma)\mathbf{p} + \mathbf{d} \tag{1}$$

Here, the rotation angles α, β, and γ indicate rotational errors in pitch, roll, and yaw, respectively, in radian. The d_x, d_y, and d_z are lateral, longitudinal, and vertical translational errors, respectively, in mm. The displacement by the set up error can be written as

$$\Delta\mathbf{p} = \mathbf{p}' - \mathbf{p} = [\mathbf{R}(\alpha, \beta, \gamma) - \mathbf{I}]\mathbf{p} + \mathbf{d} \tag{2}$$

where $\mathbf{I}$ is the 3×3 identity matrix. For the CBCT Position 0 and Position 1, the rotation matrix is given by

$$\begin{aligned}
\mathbf{R}(\alpha, \beta, \gamma) &= \begin{bmatrix} 1 & 0 & 0 \\ 0 & \cos\alpha & -\sin\alpha \\ 0 & \sin\alpha & \cos\alpha \end{bmatrix} \begin{bmatrix} \cos\beta & 0 & \sin\beta \\ 0 & 1 & 0 \\ -\sin\beta & 0 & \cos\beta \end{bmatrix} \begin{bmatrix} \cos\gamma & -\sin\gamma & 0 \\ \sin\gamma & \cos\gamma & 0 \\ 0 & 0 & 1 \end{bmatrix} \\
&= \begin{bmatrix} \cos\beta\cos\gamma & -\cos\beta\sin\gamma & \sin\beta \\ \cos\alpha\sin\gamma + \cos\gamma\sin\alpha\sin\beta & \cos\alpha\cos\gamma - \sin\alpha\sin\beta\sin\gamma & -\cos\beta\sin\alpha \\ \sin\alpha\sin\gamma - \cos\alpha\cos\gamma\sin\beta & \cos\gamma\sin\alpha + \cos\alpha\sin\beta\sin\gamma & \cos\alpha\cos\beta \end{bmatrix}
\end{aligned} \tag{3}$$

in the understanding that the 3 rotations are applied in the order of pitch, roll, and yaw.

For a sufficiently small rotation by ξ, which is the case of our rotational errors, the cosine and sine functions can be approximated to

$$\begin{aligned}
\cos\xi &= 1 - \frac{\xi^2}{2!} + \frac{\xi^4}{4!} - \frac{\xi^6}{6!} + \cdots \approx 1 \\
\sin\xi &= \xi - \frac{\xi^3}{3!} + \frac{\xi^5}{5!} - \frac{\xi^7}{7!} + \cdots \approx \xi
\end{aligned} \tag{4}$$

For instance, for $\xi = 3$ degrees $= 0.05236$ radian, $\cos\xi = 0.99863$ and $(\sin\xi)/\xi = 0.99954$. It follows that

$$\mathbf{R}(\alpha,\beta,\gamma) \approx \begin{bmatrix} 1 & -\gamma & \beta \\ \gamma+\alpha\beta & 1-\alpha\beta\gamma & -\alpha \\ \alpha\gamma-\beta & \alpha+\beta\gamma & 1 \end{bmatrix} \tag{5}$$

(Hereafter the ordinary equal sign instead of the approximate equal sign is used for simplicity.) Then, the displacement vector in Eq. (2) can be written as

$$\Delta\mathbf{p} = \begin{bmatrix} 0 & -\gamma & \beta \\ \gamma+\alpha\beta & -\alpha\beta\gamma & -\alpha \\ \alpha\gamma-\beta & \alpha+\beta\gamma & 0 \end{bmatrix} \mathbf{p} + \mathbf{d} \tag{6}$$

The displacement in each orthogonal direction is:

$$\Delta p_x = -\gamma p_y + \beta p_z + d_x \tag{7}$$

$$\Delta p_y = (\gamma+\alpha\beta)p_x - \alpha\beta\gamma p_y - \alpha p_z + d_y \tag{8}$$

$$\Delta p_z = (\alpha\gamma-\beta)p_x + (\alpha+\beta\gamma)p_y + d_z \tag{9}$$

The displacement vector length is given by

$$\|\Delta\mathbf{p}\|_2 = \sqrt{\Delta p_x^2 + \Delta p_y^2 + \Delta p_z^2} \tag{10}$$

A.2 The distribution of displacements for a group of patients

For a group of patients, the distribution of Δp_i ($i = x$, y, or z) and $\|\Delta\mathbf{p}\|_2$ can be derived with certain assumptions. Let us assume that the translational and rotational errors in all directions are independent of each other and follow normal distributions. A normal distribution can be also assumed for Δp_i which adds the translational and rotational errors (Chang 2017 and Chang 2018). Furthermore, we consider the individual point $\mathbf{p}$ that is randomly distributed on a spherical surface distanced from the origin by r. Since the expected value of the product of 2 or 3 independent random variables is zero, the variances of Δp_i can be written as

$$\overline{\Delta p_x^2} = \overline{\gamma^2}\,\overline{p_y^2} + \overline{\beta^2}\,\overline{p_z^2} + \overline{d_x^2} \tag{11}$$

$$\overline{\Delta p_y^2} = \left(\overline{\gamma^2} + \overline{\alpha^2}\,\overline{\beta^2}\right)\overline{p_x^2} + \left(\overline{\alpha^2}\,\overline{\beta^2}\,\overline{\gamma^2}\right)\overline{p_y^2} + \overline{\alpha^2}\,\overline{p_z^2} + \overline{d_y^2} \tag{12}$$

$$\overline{\Delta p_z^2} = \left(\overline{\alpha^2}\,\overline{\gamma^2} + \overline{\beta^2}\right)\overline{p_x^2} + \left(\overline{\alpha^2} + \overline{\beta^2}\,\overline{\gamma^2}\right)\overline{p_y^2} + \overline{d_z^2} \tag{13}$$

where the bar over a squared variable indicates the expected value. The point $\mathbf{p}$ in a spherical coordinate is given by $p_x = r\sin\theta\cos\phi$, $p_y = r\sin\theta\sin\phi$, and $p_z = r\cos\theta$, where $\theta \in [0,\pi]$

and $\phi \in [0, 2\pi)$. Then, the variance of p_i can be calculated by

$$\begin{aligned}
\overline{p_x^2} &= \frac{1}{4\pi r^2}\int_0^{2\pi}\int_0^{\pi} r^2 \sin\theta \left(r\sin\theta\cos\phi\right)^2 d\theta d\phi = \frac{r^2}{3} \qquad (14)\\
\overline{p_y^2} &= \frac{1}{4\pi r^2}\int_0^{2\pi}\int_0^{\pi} r^2 \sin\theta \left(r\sin\theta\sin\phi\right)^2 d\theta d\phi = \frac{r^2}{3}\\
\overline{p_z^2} &= \frac{1}{4\pi r^2}\int_0^{2\pi}\int_0^{\pi} r^2 \sin\theta \left(r\cos\theta\right)^2 d\theta d\phi = \frac{r^2}{3}
\end{aligned}$$

where the same variances in all 3 directions indicate spherical symmetry. It follows that

$$\begin{aligned}
\overline{\Delta p_x^2} &= \frac{r^2}{3}\left(\overline{\beta^2} + \overline{\gamma^2}\right) + \overline{d_x^2} \qquad (15)\\
\overline{\Delta p_y^2} &= \frac{r^2}{3}\left(\overline{\alpha^2} + \overline{\gamma^2} + \overline{\alpha^2}\;\overline{\beta^2} + \overline{\alpha^2}\;\overline{\beta^2}\;\overline{\gamma^2}\right) + \overline{d_y^2} \qquad (16)\\
\overline{\Delta p_z^2} &= \frac{r^2}{3}\left(\overline{\alpha^2} + \overline{\beta^2} + \overline{\alpha^2}\;\overline{\gamma^2} + \overline{\beta^2}\;\overline{\gamma^2}\right) + \overline{d_z^2} \qquad (17)
\end{aligned}$$

If Δp_i follows a normal distribution and the standard deviations (or the variances) are the same (i..e, $\overline{\Delta p_x^2} = \overline{\Delta p_y^2} = \overline{\Delta p_z^2}$), the vector length, $\|\Delta\mathbf{p}\|_2$, would follow a χ-distribution as described in the following subsection. However, an analytical distribution function is not generally available. Therefore, we adopted a numerical solution (a bootstrapping) to determine a percentile of $\|\Delta\mathbf{p}\|_2$.

For the CBCT Position 2, the rotation matrix is given by

$$\begin{aligned}
\mathbf{R}(\alpha,\beta,\gamma) &= \begin{bmatrix}\cos\beta & 0 & \sin\beta\\ 0 & 1 & 0\\ -\sin\beta & 0 & \cos\beta\end{bmatrix}\begin{bmatrix}1 & 0 & 0\\ 0 & \cos\alpha & -\sin\alpha\\ 0 & \sin\alpha & \cos\alpha\end{bmatrix}\begin{bmatrix}\cos\gamma & -\sin\gamma & 0\\ \sin\gamma & \cos\gamma & 0\\ 0 & 0 & 1\end{bmatrix} \qquad (18)\\
&= \begin{bmatrix}\cos\beta\cos\gamma + \sin\alpha\sin\beta\sin\gamma & \cos\gamma\sin\alpha\sin\beta - \cos\beta\sin\gamma & \cos\alpha\sin\beta\\ \cos\alpha\sin\gamma & \cos\alpha\cos\gamma & -\sin\alpha\\ \cos\beta\sin\alpha\sin\gamma - \cos\gamma\sin\beta & \sin\beta\sin\gamma + \cos\beta\cos\gamma\sin\alpha & \cos\alpha\cos\beta\end{bmatrix}
\end{aligned}$$

The Eqs. (15)-(17) corresponding to this matrix can be derived as

$$\begin{aligned}
\overline{\Delta p_x^2} &= \frac{r^2}{3}\left(\overline{\beta^2} + \overline{\gamma^2} + \overline{\alpha^2}\;\overline{\beta^2} + \overline{\alpha^2}\;\overline{\beta^2}\;\overline{\gamma^2}\right) + \overline{d_x^2} \qquad (19)\\
\overline{\Delta p_y^2} &= \frac{r^2}{3}\left(\overline{\alpha^2} + \overline{\gamma^2}\right) + \overline{d_y^2} \qquad (20)\\
\overline{\Delta p_z^2} &= \frac{r^2}{3}\left(\overline{\alpha^2} + \overline{\beta^2} + \overline{\alpha^2}\;\overline{\gamma^2} + \overline{\beta^2}\;\overline{\gamma^2}\right) + \overline{d_z^2} \qquad (21)
\end{aligned}$$

A.3 Comparison with the previous study

Chang (2017) and Chang (2018) assumed that the standard deviations (or variances) of the translational and rotational errors are the same in all directions. Then, each set of the

translational and rotational variances can be represented by a single variance: $\overline{\alpha^2} = \overline{\beta^2} = \overline{\gamma^2} \rightarrow \overline{\delta^2}$; $\overline{d_x^2} = \overline{d_y^2} = \overline{d_z^2} \rightarrow \overline{s^2}$. It follows that

$$\overline{\Delta p_x^2} = \frac{r^2}{3}\left(2\overline{\delta^2}\right) + \overline{s^2} \tag{22}$$

$$\overline{\Delta p_y^2} = \frac{r^2}{3}\left(2\overline{\delta^2} + \overline{\delta^2}\ \overline{\delta^2} + \overline{\delta^2}\ \overline{\delta^2}\ \overline{\delta^2}\right) + \overline{s^2} \tag{23}$$

$$\overline{\Delta p_z^2} = \frac{r^2}{3}\left(2\overline{\delta^2} + 2\overline{\delta^2}\ \overline{\delta^2}\right) + \overline{s^2} \tag{24}$$

For small rotational errors that make the 2nd and 3rd order terms of $\overline{\delta^2}$ negligible,

$$\overline{\Delta p_i^2} = \frac{2}{3} r^2 \overline{\delta^2} + \overline{s^2} \qquad i = x, y, \text{ or } z \tag{25}$$

This equation can be rewritten in terms of the standard deviations of rotation, translation, and their combination (σ_R, σ_S, and σ_E, respectively):

$$\sigma_E = \sqrt{\sigma_R^2 + \sigma_S^2} \tag{26}$$

where $\sigma_E = \sqrt{\overline{\Delta p_i^2}}$, $\sigma_R = \sqrt{(2/3) r^2 \overline{\delta^2}}$, and $\sigma_S = \sqrt{\overline{s^2}}$.

The displacement Δp_i scaled by its standard deviation,

$$\Delta p_i^* = \frac{\Delta p_i}{\sigma_E}, \tag{27}$$

follows the standard normal distribution, and subsequently the vector length of the normalized displacements, $\|\Delta \mathbf{p}^*\|_2 = \|\Delta \mathbf{p}\|_2 / \sigma_E$, follows a χ-distribution. Therefore, the PTV margin determined by the 100×(1-ρ)th percentile of $\|\Delta \mathbf{p}\|_2$ can be calculated by

$$M_E = \chi_\rho \sigma_E = \chi_\rho \sqrt{\sigma_R^2 + \sigma_S^2} \tag{28}$$

where χ_ρ is the critical value of the χ-distribution with 3 degrees of freedom for a significance level ρ. For instance, if $\rho = 0.05$ (i.e., 95th percentile), $\chi_\rho = 2.80$. Equation (28) is equivalent to the equation (5) in Chang (2018). We have confirmed that our bootstrapping is consistent with Eq. (28) when the abovementioned assumptions on equal variances in all directions and negligible 2nd and 3rd order terms of rotational errors are satisfied.
